# Assessment of Nasality in Adult Patients with Partial Deafness

**DOI:** 10.3390/jcm14176105

**Published:** 2025-08-29

**Authors:** Karol Myszel, Agata Szkiełkowska

**Affiliations:** 1 Center of Hearing and Speech, 7 Mokra Street, 05-830 Kajetany, Poland; 2 Faculty of Health Sciences, University of Applied Sciences, 4 Popieluszko Street, 62-510 Konin, Poland; 3 Institute of Physiology and Pathology of Hearing, 17 Mokra Street, 05-830 Kajetany, Poland; a.szkielkowska@ifps.org.pl

**Keywords:** partial deafness, nasality, cochlear implantation

## Abstract

The basic tone of the human voice is generated in the larynx, which is reinforced by and derives its distinctive features from the resonance of the oral and nasal cavities An inappropriate ratio between oral and nasal resonance results in a more nasal timbre of the voice, which is referred to as nasality (hypernasality). Nasality is often present in hearing-impaired patients, and various studies have shown that hypoacusis, including partial deafness (PD), causes voice disorders as a result of disturbed control over the complex process of voice production. This study describes our investigation of nasality in 20 adult Polish patients with post-lingual partial deafness. The results show that PD patients developed more nasality in their voices when compared with individuals in the control group. Observations made 9 months after cochlear implantation for partial deafness indicated a reduction in nasality, with the changes in acoustic parameters achieving statistical significance. **Background/Objectives**: This study aimed to assess whether partial deafness (PD) causes changes in nasal resonance in adult patients and whether partial deafness cochlear implantation (PDCI) influences the level of nasality. **Methods**: Voice samples from 20 patients attending the Institute of Physiology and Pathology of Hearing in Warsaw with partial deafness were analyzed and compared with samples from 20 individuals with normal hearing. Voice samples from the same patients were comparatively analyzed at 9 months after cochlear implantation. The level of nasality was assessed using the FFT (Fast Fourier Transform) for acoustic analysis, as well as subjective description by two experienced medical professionals (a medical doctor and a clinical acoustician). Pearson analysis was then performed to determine the correlations between the objective and subjective assessments. Paired two-sample t-tests for means were conducted for statistical analysis. All patients of the Institute of Physiology and Pathology of Hearing in Warsaw declared their deliberate consent to all necessary diagnostic and therapeutic procedures upon admittance. **Results**: The results show that post-lingual partial deafness causes nasality in adult patients when measured both objectively (*p* = 0.0001) and subjectively. The average objective level of nasality was 21 dB (SD 4.5), while the subjective level was an average grade of 1.25. The level of nasality presented a positive correlation with the duration of partial deafness. The assessment performed 9 months after cochlear implantation showed a reduction in nasality, achieving 17 dB (SD 4.2) in the objective measurement (*p* = 0.0002) and a grade of 0.5 when assessed subjectively. Pearson analysis showed a weak correlation between the objective measurement and subjective assessment (r = 0.2). **Conclusions**: Post-lingual partial deafness causes nasality in adults in a manner that is positively correlated with the duration of hearing impairment. Partial deafness cochlear implantation reduced nasality after 9 months of observation, as shown both objectively (MDVP) and subjectively (perceptual assessment). However, the correlation between the objective and subjective results is rather weak; therefore, objective acoustic methods (e.g., MDVP) should preferably be used for a more credible assessment, while the subjective method may only serve as a rough and general tool in everyday clinical use.

## 1. Introduction

Nasality is a form of disturbed resonance in speech. In normal conditions, air exhaled during speech is utilized to produce vowels when the space between the pharynx and nasal cavity is closed. Appropriate closing takes place due to the action of the velopharyngeal muscle [1,2,3]. Some medical conditions may lead to inappropriate function of this muscle, enabling airstream leakage from the oral cavity into the nose. The sound created under such conditions is perceived as open nasality (rhinolalia aperta). This phenomenon is mostly observed, to various degrees, in individuals with inborn orofacial congenital defects, complete or submucous palate cleft, short palate, or acquired conditions (e.g., scars after surgeries or radiotherapy and fistulas). In other cases, incomplete velopharyngeal closure may be caused by palate immobility associated with pathologies of the central nervous system involving the cranial nerves or certain infectious diseases. In such conditions, measurement of the energy emitted during speech demonstrates an increased ratio of nasal resonance to the sum of oral and nasal resonances (nasalence), which is subjectively perceived as nasality (hypernasality).

The production of speech is strictly controlled by the central nervous system in conjunction with hearing, which is referred to as auditory control of the voice and speech. This complex process takes place as a result of cooperation between the laryngeal muscles, central mechanisms enabling self-regulation of the amplitude, frequency, rhythm, and timbre of the voice; and the biomechanics of the abdomen, chest, and muscles of the oral cavity, including the palate. When the process of auditory control is disturbed by hearing impairment, the function of the velopharyngeal muscle may be incomplete, leading to nasality of the voice. Numerous publications in the literature have described the mechanisms of nasality in hearing-impaired individuals.

Assessments of nasality can be subjective or objective. Subjective assessments are perceptual and, in clinical practice, are usually performed by experienced phoniatricians, audiologists, or speech therapists. The perceptual assessment is conducted by an experienced medical practitioner, who listens to voice samples and compares them to healthy voices. The purpose of such an assessment is to judge the presence of excessive nasal resonance and describe its severity on a four-point scale. Objective measures include aerodynamic methods, such as nasometry and rhinospirometry, and acoustic methods, such as spectrography of the vowel “i” (SPG) and Fast Fourier Transform (FFT). Such methods are typically used to analyze the harmonic structure of the vowel “i”. According to Cudejko et al. [4], analysis of the vowel “i” is optimal for describing the level of nasality. The normal spectrogram of “i” includes two well-marked high-energy stripes; the first is formant F1 (reference value for Polish adults is 269 Hz), while the second is formant F2 (reference value for Polish adults is 2287 Hz) [5]. When nasality is present, the first formant is shifted toward higher frequencies (up to 500 Hz), causing the vowel to sound nasal. Based on analysis of the formants and average energy of the third, fourth, and fifth harmonics during the phonation of “i,” three grades of nasality were described by the authors: grade 1 (12–19 dB SPL), grade 2 (20–25 dB SPL), and grade 3 (25 dB SPL and higher). Values lower than 12 dB are considered to indicate no nasality. This scale was used as a reference for the analyses performed in later parts of this study.

## 2. Materials and Methods

**Figure 1:**
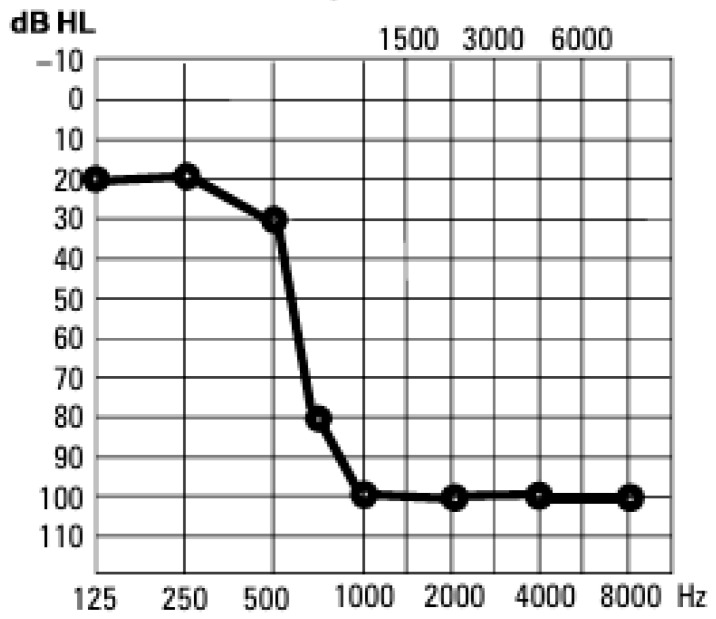
Example of pure tone audiometry results for an individual with partial deafness.

This study included 20 adult Polish patients (10 females and 10 males) with post-lingual partial deafness (PD, normal hearing thresholds up to 0.5–1 kHz and deep hearing loss for higher frequencies, as defined by Skarzynski) and 20 individuals with normal hearing as a control group. Figure 1 presents an example of the pure tone audiometry results for an individual with partial deafness. The average age of the PD patients was 50 years (SD 16.1 years), while the average age was 48 years in the control group (SD 16.4 years). Patients were selected in order to avoid outliers and ensure homogeneity with regard to both age and the duration of hearing loss. The average time of partial deafness in the study group was 19 years (SD 9.7 years). All individuals in the study group were patients of the Institute of Physiology and Pathology of Hearing in Warsaw and were qualified for partial deafness cochlear implantation. Sudden hearing loss and noise exposure were etiological factors identified to have caused post-lingual partial deafness in the study group. Only patients that were fluent in Polish and for which PD was the only reason for nasality were included in the study. Other factors, such as education level, speech habits, or dialectal background, were not considered.

The study group was limited to 20 adult individuals after careful analysis of pre-existing conditions and excluding other reasons for nasality. Many patients of the Institute of Physiology and Pathology of Hearing in Warsaw considered for partial deafness cochlear implantation were found to have co-existing pathologies, which ultimately limited the number of subjects in the study group.

Patients were subject to otolaryngological examination, including a detailed inspection of the oral cavity and palate, otoscopy, anterior and posterior rhinoscopy, and nasofiberoscopy. The audiological examination included pure tone audiometry, impedance audiometry, otoacoustic emissions, and brainstem-evoked response audiometry. Individuals were selected in such a way as to eliminate exclusion criteria, including other pre-existing conditions that might lead to nasality (palate cleft, inborn or acquired orofacial defects, neurological disorders, nasal turbinate hypertrophy, nasal polyps, nasopharyngeal tumors, allergic rhinitis, nasal congestion, subclinical velopharyngeal function). Every individual was also subject to a perceptual assessment of nasality performed by an experienced doctor and a clinical acoustician, who listened to voice samples and compared them to healthy voice samples obtained from the control group to judge the presence and severity of excessive nasal resonance. A four-point scale was used (0, none; 1, mild; 2, moderate; 3, severe), and the inter-rater reliability was 84%.

Acoustic analysis of the voice was performed with an MDVP (multi-dimensional voice program model 5105 by KayPENTAX, a Division of PENTAX Medical Company Lincoln Park, NJ 07035-1488, USA), as part of a standard procedure carried out for patients qualified for cochlear implantation in which subjects are asked to phonate the vowel “i.” Recordings of voice samples were made in an anechoic chamber before cochlear implantation and 9 months after cochlear implant activation. Processing of voice samples with the MDVP included signal analysis as a function of time and frequency. A Fast Fourier Transform (FFT) was performed to analyze and present the average amplitudes of various frequencies. The values of the analyzed amplitudes were then calculated in decibels of speech pressure levels (dB SPL), which enabled the assignment of a certain degree of nasality. The control group consisted of individuals with no nasality present (0), according to both the perceptual and objective assessments. Statistical analysis was performed using a paired two-sample t-test for means, and correlations were analyzed with the Pearson test for correlation analysis.

## 3. Results

The comparison of the signal parameters achieved in PD patients before cochlear implantation with those in the control group showed a statistically significant increase in the nasality level (*p* = 0.0001; Cohen’s d = 2.74). PD patients presented nasality of different degrees, while the control group individuals presented no nasality in their voices. The reference scale for objective nasality levels was: grade 0 (below 12 dB SPL), grade 1 (12–19 dB SPL), grade 2 (20–25 dB SPL), and grade 3 (25 dB SPL and higher). In the objective analysis, 10 subjects in the PD group presented nasality of degree 1 (50%), 6 individuals presented degree 2 (30%), and 4 individuals presented degree 3 (20%). The average nasality level was 21 dB (SD 4.5). A Pearson test was then performed to determine the correlation between nasality and the time of partial deafness. The R index reached a value of 0.33, indicating a positive correlation. Perceptual assessment revealed no nasality in 2 (10%), grade 1 in 11 (55%), grade 2 in 7 (35%), and grade 3 in none of the individuals.

**Figure 2:**
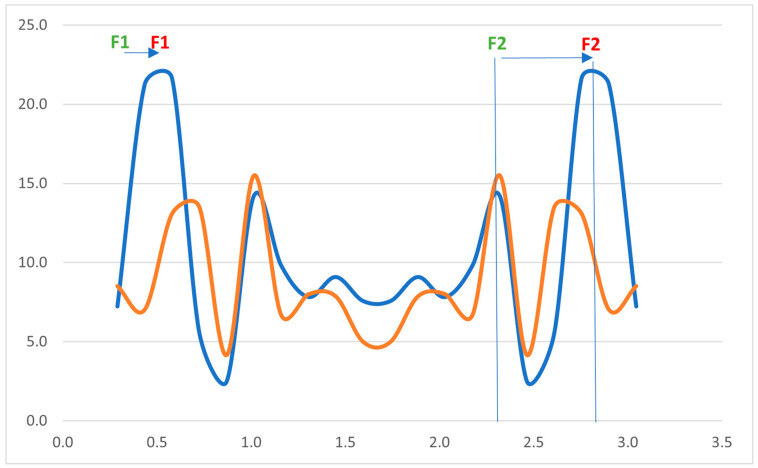
FFT analysis—average amplitude (*Y*-axis) as a function of frequency (*X*-axis) before (blue line) and after (orange line) cochlear implantation. The first formant (F1) and second formant (F2, red) are marked to show the shift toward higher frequencies vs. reference formant frequency values (green) for the vowel “i” in adults.

All patients underwent partial deafness cochlear implantation (PDCI). An analysis performed 9 months later showed an overall reduction in nasality, achieving no nasality in 2 individuals (10%), grade 1 in 14 individuals (70%), and grade 2 in 4 individuals (20%). The average nasality level was 17 dB (SD 4.2). The changes in comparison with the results before the PDCI were statistically significant (*p* = 0.0002, Cohen’s d = 0.88). The perceptual assessment also revealed a reduction in nasality: grade 0 was found in 11 (55%), grade 1 in 8 (40%), grade 2 in 1 (5%), and grade 3 in none of the individuals. Figure 2 shows the results of the Fast Fourier Transform (FFT) analysis, which demonstrates the spread of average amplitudes of certain frequencies before and after PDCI.

Individuals in the study and control groups did not present statistical differences regarding age.

**Figure 3:**
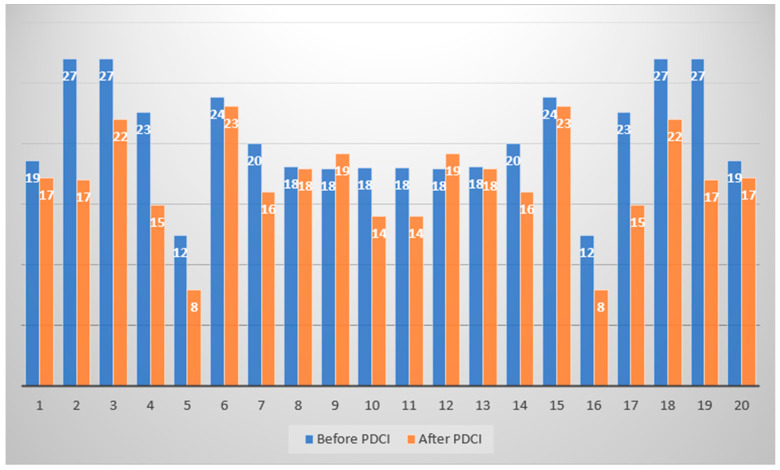
Levels of nasality (in decibels) in PD patients before and 9 months after PDCI.

The levels of nasality (in decibels) in PD patients before and 9 months after partial deafness cochlear implantation are presented in Figure 3.

In the next step, an analysis was performed to determine whether correlations exist between the subjective and objective assessments of nasality before and after PDCI. The analysis showed that the subjective (perceptual) assessment correlated weakly with the objective analysis, achieving r = 0.29 before and r = 0.20 after cochlear implantation. This weak correlation is apparently a result of the comparison made between objective measurements (i.e., computational analyses performed under standardized conditions) and perceptual assessments informed by human hearing, which may be subjectively biased. The comparison of the subjective and objective assessments of nasality before and after PDCI is presented in Table 1.

## 4. Discussion

The results show that increased nasal resonance occurs in patients with partial deafness. Disturbed auditory feedback during voice production causes inappropriate function of the velopharyngeal muscle, leading to nasality. Through the restoration of hearing control, partial deafness cochlear implantation improves voice quality and reduces nasality.

The first observations on nasality in people with hearing impairments were made years ago. The first publications on the topic were made by Hudgins and Numbers in 1942, Boone in 1966, Nober in 1967, Colton and Cooker in 1968, and Norman in 1973 [6,7,8,9]. A dependency was found between nasality and velopharyngeal function. In 1969, McClumpha [10] first observed changes in the function of the velopharyngeal muscle between subjects with normal hearing and those with hearing impairments. In all hearing-impaired subjects in the study, some velopharyngeal opening was present while producing repeated consonant–vowel syllables. A study performed by Seaver, Andrews, and Granata in 1980 [11] revealed hypernasality in 19 of 26 deaf individuals that they examined, which was associated with velopharyngeal opening. Radiological examinations excluded any anatomical deficiencies; therefore, the changes were of a typically functional character. Similar findings were reported by Stevens et al. in 1976 [8], who also found that velopharyngeal insufficiency is caused by inappropriate timing of closing and opening of the palatopharyngeal space. Another suggestion was made: that a reduced rate of speech in hearing-impaired individuals contributes to the perception of nasality, as well as an increase in the amount of air used per syllable.

Similar findings have been presented in other studies conducted in many centers, using both subjective and objective methods of nasality assessment. Ysunza and Vazques [12] examined a group of deaf individuals and observed a discoordination of velopharyngeal valving activity, despite normal muscle activity measured using electromyography. They concluded that the velopharyngeal disorder in such cases is purely functional and results from inappropriate auditory regulation during phonation. Fletcher, Mahfuzh, and Hendarmin [13] investigated nasality in a group of hearing-impaired children and compared them with a group of their normal-hearing peers. They showed that children with hearing loss presented more nasality, in accordance with the results achieved in our study.

Further studies, such as those of Baudonck et al. and Swapna et al. [14], have proven that cochlear implants improve nasality to a greater degree than traditional hearing aids; however, the level of nasality in both cases was still higher than that in normal-hearing individuals. Our study also demonstrated a decrease in the level of nasality in patients after cochlear implantation.

Chen [15] discovered that acoustic analysis of speech in hearing-impaired children presents reduced first-formant prominence and a correlation with perceptual assessments of nasality. They also proved the usefulness of acoustic voice analysis in the assessment of resonance. In their research, Kim et al. [16] found that a normal-hearing group of subjects demonstrated significantly lower nasality than a hearing-impaired group. They also found that the hearing-impaired subjects presented a tendency toward velopharyngeal opening during the phonation of vowels. Sebastian et al. [17] reported an improvement in nasality after cochlear implantation. Analogous conclusions were drawn in the studies conducted by Nguyen et al. [18] and Guillot et al. [19].

The study described in this article led to similar conclusions. Partial deafness, as a hearing impairment with appropriate hearing thresholds in low frequencies and deep hearing loss at high frequencies, was found to cause nasality in the affected patients. The statistically significant reduction in the level of nasality after cochlear implantation proves that restoring hearing control of the voice leads to a reduction in nasal resonance, improving the mechanisms of voice production. The study also confirmed that acoustic methods are a valid tool for the objective measurement of nasality, providing more consistent results than perceptual assessments.

The study has some limitations. One is the small number of patients in the study group; therefore, further studies in bigger study groups should be conducted to validate the presented results. Long-term analyses (e.g., over several years following cochlear implantation) would also be valuable to observe whether further improvements in voice quality take place and whether the reduction in nasality is stable and continuous.

## 5. Conclusions

This study showed that partial deafness causes nasality in adults with post-lingual partial deafness. The apparent reason for such nasality is a disturbance of the central control mechanisms of voice production, which are normally coordinated with hearing. Pearson analysis revealed that nasality is positively correlated with the duration of partial deafness.

Partial deafness cochlear implantation led to a reduction in nasality after 9 months, which is considered to be due to the restoration of auditory control over the voice. Aside from improved hearing, patients also benefit from a reduction in nasality as a result of partial deafness cochlear implantation, which may improve their quality of life.

## Figures and Tables

**Table 1:** Grades of nasality in the subjective and objective assessments of PD patients before and 9 months after cochlear implantation.

Grade of Nasality	Before PD Cochlear Implantation				After PD Cochlear Implantation			
	Perceptual Assessment	Percentage	Objective Assessment	Percentage	Perceptual Assessment	Percentage	Objective Assessment	Percentage
0	2	10%	0	0%	11	55%	2	10%
1	11	55%	10	50%	8	40%	14	70%
2	7	35%	6	30%	1	5%	4	20%
3	0	0%	4	20%	0	0%	0	0%
	Correlation r = 0.29				Correlation r = 0.20			

## Data Availability

The data presented in this study are available from the corresponding author upon request.
